# A novel antifungal protein of *Bacillus subtilis* B25

**DOI:** 10.1186/2193-1801-2-543

**Published:** 2013-10-17

**Authors:** Zhiqiong Tan, Baoying Lin, Rongyi Zhang

**Affiliations:** Key Laboratory of Protection and Development Utilization of Tropical Crop Germplasm Resources, Hainan University, 58#, Renmin road, 570228 Haikou, Hainan Province China

**Keywords:** *Bacillus subtilis*, Antifungal protein, Purification

## Abstract

*Bacillus subtilis* B25 was isolated from banana rhizosphere soil. It has been confirmed for B25 to have stronger antagonism against *Fusarium oxysporum* f.sp*.cubense*, Additionally B25 has good inhibitory to plant pathogens, including *Corynespora cassiicola*, *Alternaria solani*, *Botrytis cinerea* and *Colletotrichum gloeosporioides* on potato dextrose agar (PDA) plates. The antagonistic substance can be extracted from cell-free culture broth supernatants by 70% (w/v) (NH4)2 SO4 saturation. Clear blank band was observed between the protein and a pathogen. The examination of antagonistic mechanism under light microscope showed that the antifungal protein of B25 appeared to inhibit pathogens by leading to mycelium and spores tumescence, distortion, abnormality. The isolation procedure comprised ion exchange chromatography on DEAE-Sephadex Fast Flow and gel filtration chromatography on SephadexG-100. The purified antifungal fraction showed a single band in sodium dodecyl sulfate-polyacrylamide gel electrophoresis (SDS-PAGE). The active fraction was identified by NanoLC-ESI-MS/MS The amino acid sequences of 17 peptides segments were obtained. The analysis of the protein suggested that it was a hypothetical protein (gi154685475), with a relative molecular mass of 38708.67 Da and isoelectric point (pI) of 5.63.

## Introduction

*Fusarium* wilt, caused by *Fusarium oxysporum* f.sp*.cubense* (FOC.), was a devastating disease of banana, which was threatening the safety of banana production in China and the worldwide. At present, this disease have spread to a very large of area. Effective control methods for it are not available. Banana as one of the most important economic crops, its contribution to the overall fruit production reached a very high level, but now many existing banana orchard is being wiped out. It was a potential measure to screen antagonistic bacteria and produce microbial germicide in the control of *Fusarium* wilt of banana. The chemical control and use of fungicides are the most effective way of preventing the occurrence of plant diseases. However, the use of chemicals is considered undesirable because of concerns over residues, following an increased public health concern. The bio control has become an interesting alternative to conventional methods.

*B.subtilis*, which distribute in the nature widely as non-pathogenic bacteria, can nonribosomally synthesizes several kinds of small antibiotic peptides (<2000 Da) that have antifungal activities, such as iturin (Delcambe et al. 1977; Tsuge et al. 2001; Stein 2005), surfactin (Peypoux et al. 1999; Carrillo et al. 2003), fengymycin, bacilysin (Loeffler et al. 1986), bacillomycin (Peypoux et al. 1980), mycosubtilin (Peypoux et al. 1986), and mycobacillin (Sengupta et al. 1971). Additionally *B. subtilis* secretes an abundance of proteins (Liu et al. 2007; Chen *et al*. 2010; Luo et al. 2013).

*Bacillus subtilis* B25 was isolated from banana rhizosphere soil in Hainan. It has been confirmed for B25 that antagonism against *Fusarium oxysporum* f.sp.*cubense* (FOC) and other plant fungal pathogen can be produced (Liu 2011, China). We found that the antimicrobial substance could tolerate acid and not changed significantly even in 100°C boil 1 hour (Lin 2013, China). The aim of this paper was to extract the antifungal substances of *Bacillus subtilis* B25 and test it’s activity.

## Materials and methods

### Chemicals

All chemicals were analytical grade. Bovine serum albumin was from Sigma. Low-molecular weight protein standards were purchased from TaKaRa Biotechnology (Dalin) Co., Ltd. (China), DEAE-Sephadex Fast Flow was obtained from GE, SephadexG-100 from Pharmacia.

### Microorganisms and culture conditions

*B. subtilis* B25 was grown in fermentation medium containing (g/L): glucose, 8.0; peptone, 8.0; yeast extract, 1.0; beef extract, 3.0; NaCl, 10.0; The pH of the medium was adjusted to 7.0 before autoclaving.

Cultivation was performed using 250 ml Erlenmeyer flasks containing 100 ml medium for 48 h at 28°C. As inoculation, 2% (v/v) bacteria grew in Luria-Bertani broth (g/L): peptone, 10.0; yeast extract, 5.0; and NaCl, 10.0; pH 7.0.

*Fusarium oxysporum* f.sp.*cubense* was isolated from wilted banana vascular and stored at 4°C in PDA and it was used as the indicator.

B25 was isolated from banana rhizosphere soil and identified as *Bacillus subtilis* on the basis of its morphological, biochemical and physiological characteristics, and 16S r DNA sequence. It’s capability of controlling Fusarium wilt in green house and in the field was evaluated (Liu et. al. 2011). Also, the characteristics of the active substance, such as precipitated quality, molecular mass, heat-tolerance were test (Lin et. al. 2013).

### Assay of of antifungal activity

B25 was assayed for its antagonistic activity *in vitro* against *Fusarium oxysporum* f.sp*.cubense*, *Corynespora cassiicola*, *Alternaria solani*, *Botrytis cinerea*, *Colletotrichum gloeosporioides* and *Aspergillus niger* via duel culture method. A fungal disc of five day old mycelial mat of 1 cm diameter was placed in the centre of the petri plate containing PDA (potato dextrose agar). And the 24 hr old *Bacillus* B25 culture was single streaked at a distance of approx. 3 cm from the fungal disc. The plate was incubated at 28°C and observed after every 24 hrs for any inhibition of mycelial growth.

The antifungal activity against FOC of precipitated protein and all fractions acquired in the procedure of separation was tested by agar-diffusion method. A fungal disc was placed in the centre of PDA. Plate. Wells of 0.5 cm diameter were made at a distance of approx. 3 cm from the fungal disc. And tested substances were discharged in the wells. The Plate was incubated at 28°C and observed for any inhibition of mycelial growth.

### Production and purification of antibiotics

A loop of B25 cells from a slant culture of fresh nutrient agar was inoculated to a 250 ml flask containing 100 ml LB broth (pH 7.0). The flask was incubated on a rotary shaker at 200 rpm for 9 ~ 12 h at 28°C. This fresh culture was inoculated to fermentation broth, each 2 ml. These flasks were incubated under the same conditions as described above for 48 h. Culture supernatants obtained after removed the cells by centrifugation at 6000 r/min for 20 min for further study.

The proteins were precipitated from the supernatant at 70% (w/v) (NH4)_2_ SO_4_ saturation. The latter was added in small portions with constant stirring for 30 min. The stirring was continued for 1 h, and the mixture was kept overnight at 4°C. The precipitate was collected by centrifugation at 10000 r/min for 30 min, dissolved in a 1/20 (v/v) phosphate buffer (0.02 mol/L, pH 6.8), and dialyzed for 48 h with 8 changes in the same buffer (500 ml each) to remove ammonium sulfate. The dialysates were condensed to yield precipitated proteins, which were further purified by column chromatography. A part of the precipitated proteins were dissolved in phosphate buffer (0.02 mol/L, pH 6.8), and the antifungal activity of the precipitated proteins was tested against *Fusarium oxysporum* by agar-diffusion method*.*

Chromatography was carried out on a DEAE-sepharose fast Flow ion exchange column previously equilibrated with 0.02 mol/L phosphate buffer (pH 6.8). The column was eluted with NaCl in phosphate buffer (0.02 mol/L, pH 6.8) to desorb the absorbed proteins (fractions I, II, III, IV). Each fraction was dialyzed in distilled water to remove NaCl, and then adjusted to the same concentration with phosphate buffer. Anti-fungal activity was tested against *Fusarium oxysporum* by agar- diffusion method. Fraction III was subsequently chromatographed on SephadexG-100. The column was eluted with 0.02 mol/L phosphate buffer (pH 6.8) to collect one main protein peak (P1) and another small protein peak (P2). The antifungal activity of the two fractions was tested. The protein concentration was determined using the method of Bradford (1976), with bovine serum albumin as a standard.

### NanoLC-ESI-MS/MS analysis

The antifungal protein was separated by SDS-PAGE, analyzed by NanoLC-ESI-MS/MS, (ProtTech.Inc).

## Results

### Spectrum of antifungal activity

The antifungal spectra of B25 against FOC, *Corynespora cassiicola*, *Alternaria solani*, *Botrytis cinerea*, *Colletotrichum gloeosporioides* and *Aspergillus niger*, are shown in Table 1. The results indicated that B25 had a wide spectrum of antagonistic activities against plant pathogenic fungi.

**Table 1:** **Spectrum of antifungal activity**

Plant pathogens	Inhibition zone (mm)
*Fusarium oxysporum* f.sp*.cubense*	11.5aA
*Corynespora cassiicola*	10.0dD
*Alternaria solani*	12.1bB
*Botrytis cinerea*	9.8bBC
*Colletotrichum gloeosporioides*	18.3dD
*Aspergillus niger*	12.3bB

Lowercase: p = 0.05; Capital letter: p = 0.01.

### Antifungal activity of precipitated proteins

**Figure 1:**
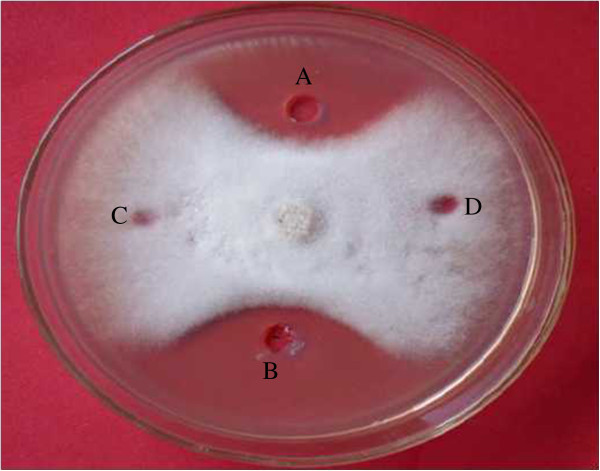
**Antifungal activity of precipitated proteins toward** ***Fusarium oxysporum.*** **A**, **B**: precipitated proteins; **C**, **D**: control (10 μl 20 mmol/L Na2HPO_4_/NaH2PO4 buffer).

Antifungal factor from B25 was further identified as a kind disease-resistance protein by ammonium sulfate precipitation method. The protein extracted could inhibit the pathogen in PDA (potato dextrose agar) medium and a clear zone of inhibition was observed between the protein and pathogen (Figure 1).

### Preliminary function of the antifungal protein

**Figure 2:**
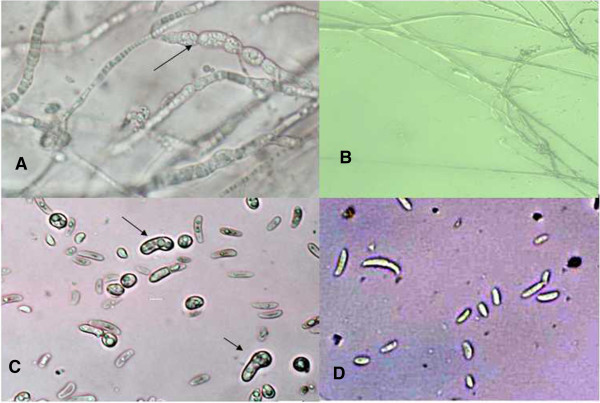
**Function of the antifungal protein. A**: Abnormal hypha appeared distorted, tumescent after treatment with; **B**: Normal hypha of FOC*;* **C**: Abnormal spores of FOC appeared distortion and tumescence after treatment with; **D**: Normal spore of *F. oxysporum*.

Inhibitory effect of precipitated proteins on FOC as seen under the light microscope demonstrated that it had inhibitory effect on mycelia and spores. The disease-resistance protein caused distortion, tumescence of hyphae and spores (Figure 2).

### Isolation and purification of antifungal protein

**Figure 3:**
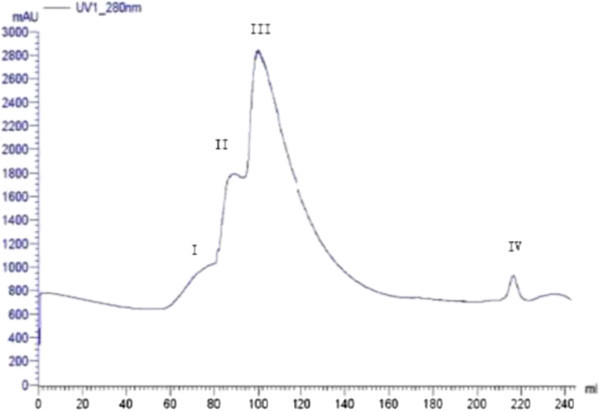
**Ion exchange chromatography on a DEAE-sepharose Fast Flow.** Sample: precipitated proteins from the supernatant in Na2HPO 4/NaH2PO4 buffer (20 mmol/L, pH6.8). Slanting line across the chromatography indicates NaCl employed to elute the absorbed fractions I, II, III, and IV.

**Figure 4:**
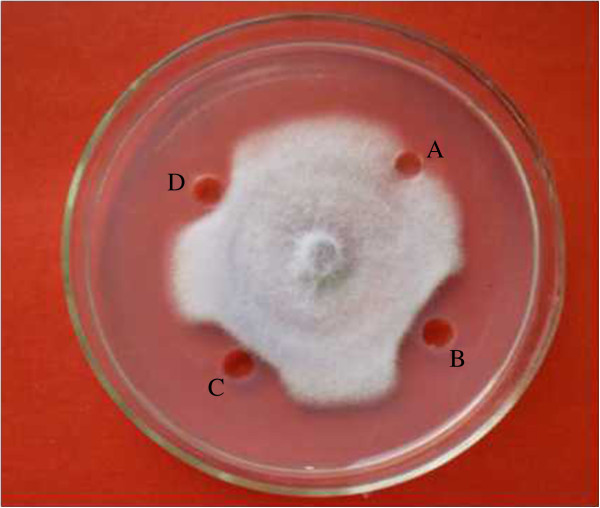
**Antifungal activity of fractions from DEAE-sepharose Fast Flow and SephadexG-100 chromatographies toward** ***Fusarium oxysporum.*** **A**: control (10 μl 20 mmol/L Na2HPO 4/NaH2PO4 buffer (PB buffer), pH 6.8); **B**: fraction III from DEAE-sepharose Fast Flow column chromatography; **C**: 20 μg antifungal protein (fraction P1) in 10 μl PB buffer; **D**: 12 μg antifungal protein (fraction P1) in 10 μl PB buffer.

**Figure 5:**
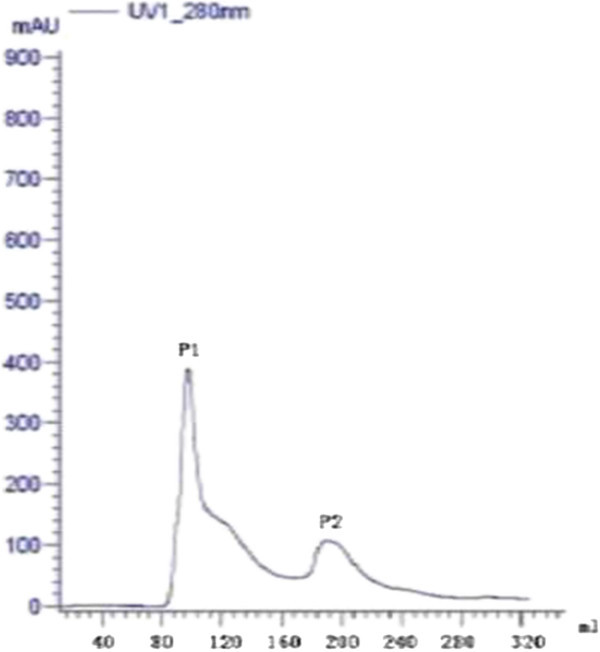
**Gel chromatography on a SephadexG-100 column.** Sample: fraction III from DEAE-sepharose Fast Flow column chromatography.

**Figure 6:**
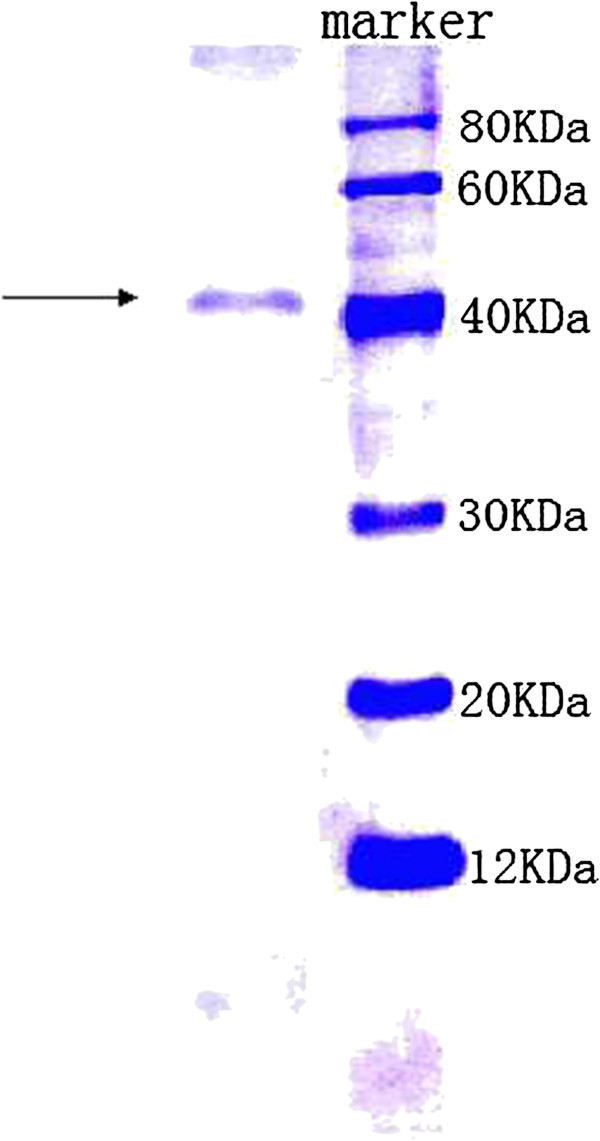
**Sodium dodecy1 sulfate-polyacrylamide gel electrophoresis of fraction P1 from SephadexG-100 column chromatography.** Arrow: Purified angifungal protein; Marker: Low molecular mass markers from TaKaRa Biotechnology (China).

Ion exchange chromatography of *B. subtilis* B25 culture extract on a DEAE-sepharose. Fast Flow column contained one unabsorbed fraction and 4 adsorbed fractions (I, II, III, and IV) (Figure 3). Of these fractions, only fraction III showed strong antifungal activity (Figure 4), and fraction II showed a less antifungal activity. Fraction III was separated on the SephadexG-100 column into two fractions (P1 and P2) (Figure 5). Only fraction P1 exhibited antifungal activity (Figure 4) and showed a single band in SDS-PAGE (Figure 6). The protein yields at various stages of chromatographic purification are shown in Table 2.

**Table 2:** **Protein yields of chromatographic fractions with antifungal activity obtained at different stages of purification**

Fraction	Total protein (mg)	Recovery of protein (%)
Precipitated protein	232.1	100.0
DEAE-sepharose Fast FlowIII	18.0	77.6
SephadexG-100P1	15.3	85

### Amino acid sequence analysis

17 peptides were obtained by NanoLC-ESI-MS/MS. The amino acid sequences of 17 peptides segments were listed in Table 3. The protein mass was 38708.67 Da, and it’s isoelectric point (pI) was 5.63. After searching the protein database of NCBI for the identity, we found that it was a hypothetical protein, which was derived from the genome of *Bacillus amyloliquefaciens* FZB42(gi 154685475). Except for the amino sequence, no any other information such as function, location, mass, and isoelectric point had ever been reported. Now, we found that it can be secreted by the bacteria, and acted on the plant fungi in the environment.

**Table 3:** **Peptides and their sequences**

Scan no.	Peptide mass	Peptide sequence
3614	865.48	HLAGLAER
3679	1247.64	TKEETSDLGIR
3794	1305.61	DASGPYHYQLR
3971	1225.67	THELSLLNTAK
4005	1629.92	KGGLFVTIPGRDDKK
4053	1501.82	KGGLFVTIPGRDDK
4067	1978.00	TYTGTILMHQTSVHVYK
4121	1373.73	GGLFVTIPGRDDK
4130	1143.68	KGGLFVTIPGR
4174	1467.81	LLTAHVDTLGAMVK
4202	1151.56	VGDFISFDPR
4252	2284.13	SGHDIVHGLIGPGIDASHAFER
4256	2284.13	SGHDIVHGLIGPGIDASHAFER
4334	1130.68	LKIDLIGGFR
4653	1790.04	HLDDKASVALLLNLIR
4672	1181.75	ASVALLLNLIR
4777	2623.36	ELVSIPSPTGNTYEVIAYTESLLK

## Conclusion

The growing awareness of preservation o f the environment and health concerns drive the search for bio-safer and environmental friendly products than chemical pesticides. Increased concerns over the impact of chemicals on the environment have resulted in increase interest in biocontrol strategies. Biological control of *Fusarium* wilt by utilizing selected antagonistic bacteria might be an alternative approach.

The novel protein may be a potential biocontrol candidate and the report of it can supply additional literature on *Bacillus subtilis* antifungal proteins which control the *fusarium* wilt of banana. Further investigations of the nature and the structure, together with development of suitable application, could have great potential for the control of plant pathogenic fungi.

## Discussion

The molecular mass of it was not only larger than any other small antibiotic peptides, but also different from those of other reported antifungal proteins from *B. subtilis*, such as Bacisubin (41.9 kDa), protein from B.s G87 (50.8 kDa), protease (41.38 kDa), bacillomycin D synthetase A (448.21 kDa). Additionally, it was demonstrated neither protease, nor ribonuclease activity. And the protein has wide antifungal spectrum, which include plant pathogens and *Aspergillus niger* The action of the protein on the bacteria was not tested.

### Ethical standards

All participants gave written informed consent. All authors have no dispute on the order of the writers.
